# Qualitative-Modeling-Based Silicon Neurons and Their Networks

**DOI:** 10.3389/fnins.2016.00273

**Published:** 2016-06-15

**Authors:** Takashi Kohno, Munehisa Sekikawa, Jing Li, Takuya Nanami, Kazuyuki Aihara

**Affiliations:** ^1^Institute of Industrial Science, University of TokyoTokyo, Japan; ^2^Department of Mechanical and Intelligent Engineering, Utsunomiya UniversityUtsunomiya, Japan; ^3^College of Electronic Engineering, Xi'an Shiyou UniversityXi'an, China; ^4^Department of Electrical Engineering and Information Systems, University of TokyoTokyo, Japan

**Keywords:** qualitative modeling, silicon neuron, non-linear dynamics, low-power circuit, neuronal network emulation

## Abstract

The ionic conductance models of neuronal cells can finely reproduce a wide variety of complex neuronal activities. However, the complexity of these models has prompted the development of qualitative neuron models. They are described by differential equations with a reduced number of variables and their low-dimensional polynomials, which retain the core mathematical structures. Such simple models form the foundation of a bottom-up approach in computational and theoretical neuroscience. We proposed a qualitative-modeling-based approach for designing silicon neuron circuits, in which the mathematical structures in the polynomial-based qualitative models are reproduced by differential equations with silicon-native expressions. This approach can realize low-power-consuming circuits that can be configured to realize various classes of neuronal cells. In this article, our qualitative-modeling-based silicon neuron circuits for analog and digital implementations are quickly reviewed. One of our CMOS analog silicon neuron circuits can realize a variety of neuronal activities with a power consumption less than 72 nW. The square-wave bursting mode of this circuit is explained. Another circuit can realize Class I and II neuronal activities with about 3 nW. Our digital silicon neuron circuit can also realize these classes. An auto-associative memory realized on an all-to-all connected network of these silicon neurons is also reviewed, in which the neuron class plays important roles in its performance.

## 1. Introduction

The nervous system allows individual animals and their populations to survive in severe environments by analyzing a huge amount of information from sensory organs and promptly generating adequate control signals for motor organs. This complex and intelligent information processing ability is autonomously obtained and adaptively maintained on its genetically developed physical basis, the network of neuronal cells. The nervous system consumes a sufficiently low power to allow for operation within the power supply limit of an animals' body; for example, the human brain consumes about 20 W (Clarke and Sokoloff, 1999), which is a lower power than mainstream CPUs. Because it is a network of neuronal cells with a wide variety of complex activities, the mechanisms of its information processing function are still poorly understood. It is attracting increased attention from biological, medical, and engineering fields.

A silicon neuronal network is a network of silicon neurons (SNs) connected via silicon synapses (SSs), which are electronic circuits that reproduce the electrophysiological activity of neuronal cells and synapses, respectively. Unlike neuro-inspired artificial neural networks, it is an approach to neuromimetic systems that realize intelligent, autonomous, robust, and power-efficient information processing via an architecture comparable to the nervous system (Arthur and Boahen, 2011; Brink et al., 2013b; Cassidy et al., 2013; Kohno et al., 2014b; Qiao et al., 2015; Giulioni et al., 2016). Because it is a bottom-up approach with cell-level granularity and reproduces neuronal spiking activities, it is also applicable to biohybrid systems including neuroprosthetic devices that replace damaged nerve tissues (Ambroise et al., 2013). Generally, SN circuits are required to have the capability of reproducing complex neuronal activities, have a low power consumption, and be compact and highly integratable.

In fields where the reproducibility is important, such as the biohybrid systems and high-speed simulators, SN circuits have been designed to solve ionic conductance neuronal models (Simoni and DeWeerth, 2006; Schemmel et al., 2010; Grassia et al., 2011; Saïghi et al., 2011). These models describe the dynamics of ionic currents that generate the dynamical behavior of the membrane potential by charging and discharging the membrane capacitance. They can precisely reproduce neuronal activities, but their equations are described by high-dimensional non-linear differential equations (DEs). It was demonstrated that their circuit implementations (conductance-based SNs) can well reproduce the neuronal activities of their target cells but require a relatively large amount of hardware resources and consume a relatively high power in the range of micro- to milliwatts. The ionic conductance models share a common structure, namely the Hodgkin–Huxley formalism, which allows their circuit implementation to mimic a variety of neuronal cells after fabrication by applying appropriate parameter voltages (Grassia et al., 2011; Saïghi et al., 2011).

In fields where low power consumption and integratability are important, SN circuits that solve integrate-and-fire (I&F) models are widely used. These models describe the neuronal activities with simple DEs by treating a spike as an event and focusing on the timing of spike generation. Their analog and digital circuit implementations (I&F-based SNs) have been developed (Thomas and Luk, 2009; Arthur and Boahen, 2011; Cassidy et al., 2013; Merolla et al., 2014; Mayr et al., 2015; Qiao et al., 2015; Giulioni et al., 2016). Analog I&F-based SNs achieve ultralow power consumption down to several nanowatts and several hundreds of them were integrated on a chip with thousands of SS circuits. Although their digital implementations consume more power, they are more portable, easy-to-operate, and highly integratable. A milestone work is the TrueNorth chip (Merolla et al., 2014) that integrates 1 million SNs and 256 million SSs on an application-specific integrated circuit chip and consumes less than 70 mW. Silicon neuronal networks implemented on field-programmable gate array (FPGA) chips achieve far less integration (about 1000 SNs) and consume higher power, but their low cost and reconfigurability have attracted many researchers. Sophisticated I&F-based models such as the Izhikevich (IZH) (Izhikevich, 2004) and adaptive exponential I&F (Brette and Gerstner, 2005) models incorporate the viewpoint of qualitative neuronal modeling described below, which allows them to reproduce a variety of neuronal activities. In principle, however, they cannot reproduce some neuronal properties related to the variability of spikes, which are reported experimentally and indicated theoretically. For example, it was reported that the amplitude of spikes at an axon terminal in the hippocampus is gradedly dependent on the stimulus intensity (Alle and Geiger, 2006), and a mathematical analysis of neuronal models indicated that a class of neurons, Class II in Hodgkin's classification (Hodgkin, 1948), can generate spikes in the same manner (Rinzel and Ermentrout, 1998). In addition, the parameter setting of the I&F-based models requires careful tuning; for example, we pointed out that the phase response curve (PRC) of the IZH model in the Class II setting is discontinuous at θ = 0, which causes a severe reduction in the retrieval ability of an auto-associative memory in all-to-all connected networks (Osawa and Kohno, 2015). This problem can be solved by increasing the parameter *d* of the model, which distorts the waveforms of the membrane potential by producing a huge hyperpolarization after each spike. Another example is that the spiking patterns of the IZH model in the intrinsic bursting (IB) setting have different characteristics from IB cells when a strong stimulus is applied (Nanami and Kohno, 2016). These facts suggest the possibility that a network of I&F-based silicon neurons may have limited ability to reproduce particular information processing in the nervous system.

In the field of qualitative neuronal modeling, the mathematical techniques of non-linear dynamics have been effectively applied to ionic-conductance models to produce low-dimensional and simple polynomial equations that qualitatively capture their dynamical properties (Rinzel and Ermentrout, 1998; Izhikevich, 2007). In contrast to the I&F approach, they do not ignore specific phenomena including the spike generation mechanism. The most well-known qualitative model is the FitzHugh–Nagumo (F-N) model (FitzHugh, 1961) that reproduces the dynamical structure in the Hodgkin–Huxley (H-H) model (Hodgkin and Huxley, 1952). The H-H model is described by four-variable non-linear DEs, whereas the F-N model is two-variable and its only non-linear term is cubic. The F-N model is Class II and can produce the graded spike response to a pulse stimulus. The first silicon nerve membrane circuit, the Nagumo circuit (Nagumo et al., 1962), implemented this model using the tunnel diode. Later, several SNs have implemented the F-N and other qualitative models using recent analog and digital circuit technologies (Linares-Barranco et al., 1991; Cosp et al., 2004; Weinstein and Lee, 2006).

In our previous works (Kohno and Aihara, 2005, 2007, 2008a; Sekikawa et al., 2008; Kohno and Aihara, 2010; Li et al., 2012; Kohno and Aihara, 2014a; Kohno et al., 2014b), we proposed a qualitative-modeling-based design approach for SNs. In this approach, a qualitative neuronal model that reproduces the dynamical structure in a target neuronal model is constructed by combining the formulae for the characteristic curves of favorable elemental circuit blocks instead of polynomials. The elemental circuit blocks are selected according to the required features of the SN; for example, subthreshold metal–oxide–semiconductor field-effect transistor (MOSFET) circuit blocks may be used for low-power SNs. Such a model is expected to be implemented efficiently in comparison to the direct implementation of polynomial-based qualitative models. In addition, a model that supports the mathematical structures in different classes of neurons can be designed, and one of them is invoked by appropriately selecting the model parameters. We developed a configurable low-power analog SN circuit (Kohno and Aihara, 2008a,b; Sekikawa et al., 2008; Kohno and Aihara, 2010) and a configurable simple digital SN circuit (Kohno and Aihara, 2007; Li et al., 2012, 2013). Our analog SN supports five classes of neuronal activities, Class I and II in the Hodgkin's classification, regular spiking (RS), square-wave bursting, and elliptic bursting (Wang and Rinzel, 2003) by appropriately setting the parameter voltages, and our digital SN supports Class I and II and Class I^*^ (Tadokoro et al., 2011) neuronal activities. Basu and Hasler (2010) developed two ultralow-power analog SNs that consume several nanowatts (Brink et al., 2013b) on the basis of a similar approach; one of them is dedicated to Class I and another to Class II. We are developing an analog SN that supports both classes and consumes a low amount of power that is comparable to their work (Kohno and Aihara, 2014a).

Most silicon neuronal networks incorporate the SS circuits that mimic the signal transmission in chemical synapses. Their three important features are the synaptic efficacy, plasticity, and summation (Destexhe et al., 1998; Song et al., 2000; Dan and Poo, 2004). A large (small) amount of synaptic current is injected into the postsynaptic neuronal cell if the synaptic efficacy is high (low). The synaptic efficacy is modulated on the basis of some factors including the neuronal spikes generated by the pre- and postsynaptic neuronal cells (the synaptic plasticity). It is called the spike-timing-dependent plasticity (STDP) if its rule (a learning rule) is based on the timing of neuronal spikes in the pre- and postsynaptic neuronal cells (Song et al., 2000; Dan and Poo, 2004). The synaptic summation allows a bursting spike input to enhance the effect of synaptic transmission. It was shown that this feature can play a critical role in spike timing recognition (Gütig and Sompolinsky, 2006). Note that the information of an input spike's magnitude can be transmitted via the time period of neurotransmitter release. The compactness and low-power consumption of SS circuits are also an important issue because the number of SSs in a silicon neuronal network is generally larger than that of SNs. In Merolla et al. (2014), the integration of a huge number of digital SSs was realized by limiting the functionality of the SS to the synaptic efficacy. Their synaptic weights have to be calculated by a off-chip system, but this is not a limitation in engineering applications in which “ready-trained” discriminators are required. They reported that this circuit could realize a multiobject detection and classification system. Only the synaptic efficacy was supported also in early FPGA-based silicon neuronal networks (Rice et al., 2009; Thomas and Luk, 2009), but in recent works, the synaptic summation is supported in Ambroise et al. (2013) and all of the three features are supported in Li et al. (2013) and Cassidy et al. (2013). The analog SS circuit in Giulioni et al. (2016, 2015) implements the synaptic efficacy and the plasticity. Their silicon neuronal network chip integrates 128 leaky I&F SNs and 16384 SSs whose synaptic efficacy is stored in a bistable memory and controlled by a Hebbian-type STDP rule (Fusi et al., 2000). They realized an autoassociative visual memory (Giulioni et al., 2015) and motion detectors (Giulioni et al., 2016). The analog SS circuit in Qiao et al. (2015) implements all of the three features of synapses. The synaptic summation is realized by a low-power current-mode integrator circuit called a differential-pair integrator (DPI). To reduce the circuit size, a DPI circuit is shared by multiple synapses by exploiting its linearity. The synaptic efficacy is stored in a bistable memory and controlled by an STDP-based algorithm (Brader et al., 2007). This chip integrates 256 adaptive exponential I&F SNs with more than 128,000 SSs and was applied to image classification tasks. Another full-featured analog SS in Brink et al. (2013b) stores the synaptic efficacy in an analog non-volatile memory based on a floating-gate device and supports an asymmetrical STDP learning rule. This chip integrates 100 Class II SNs with 30000 SSs and realized a winner-take-all network and a rhythm generator (Brink et al., 2013a).

In this article, we briefly review our SN circuits designed by a qualitative-modeling-based approach. The next section summarizes the mathematical methods of qualitative neuronal modeling that are applied to SN design. Section 3 explains our analog and digital SNs and Section 4 concludes this review.

## 2. Qualitative neuronal modeling

In spiking neuronal cells, fast ionic currents such as the fast sodium and rectifying potassium currents are responsible for spike generation. Slower ionic currents such as the calcium currents and the potassium currents that are controlled by the intracellular calcium concentration modify the dynamics of the spike generation system. Various types of neuronal cells are known and each of them has its own combination of expressed ionic channels, which leads to a variety of neuronal activities. The mechanisms of these dynamical activities have been considerably elucidated from the perspective of non-linear dynamics (Rinzel and Ermentrout, 1998; Wang and Rinzel, 2003; Izhikevich, 2007).

### 2.1. Spike generation systems

**Figure 1:**
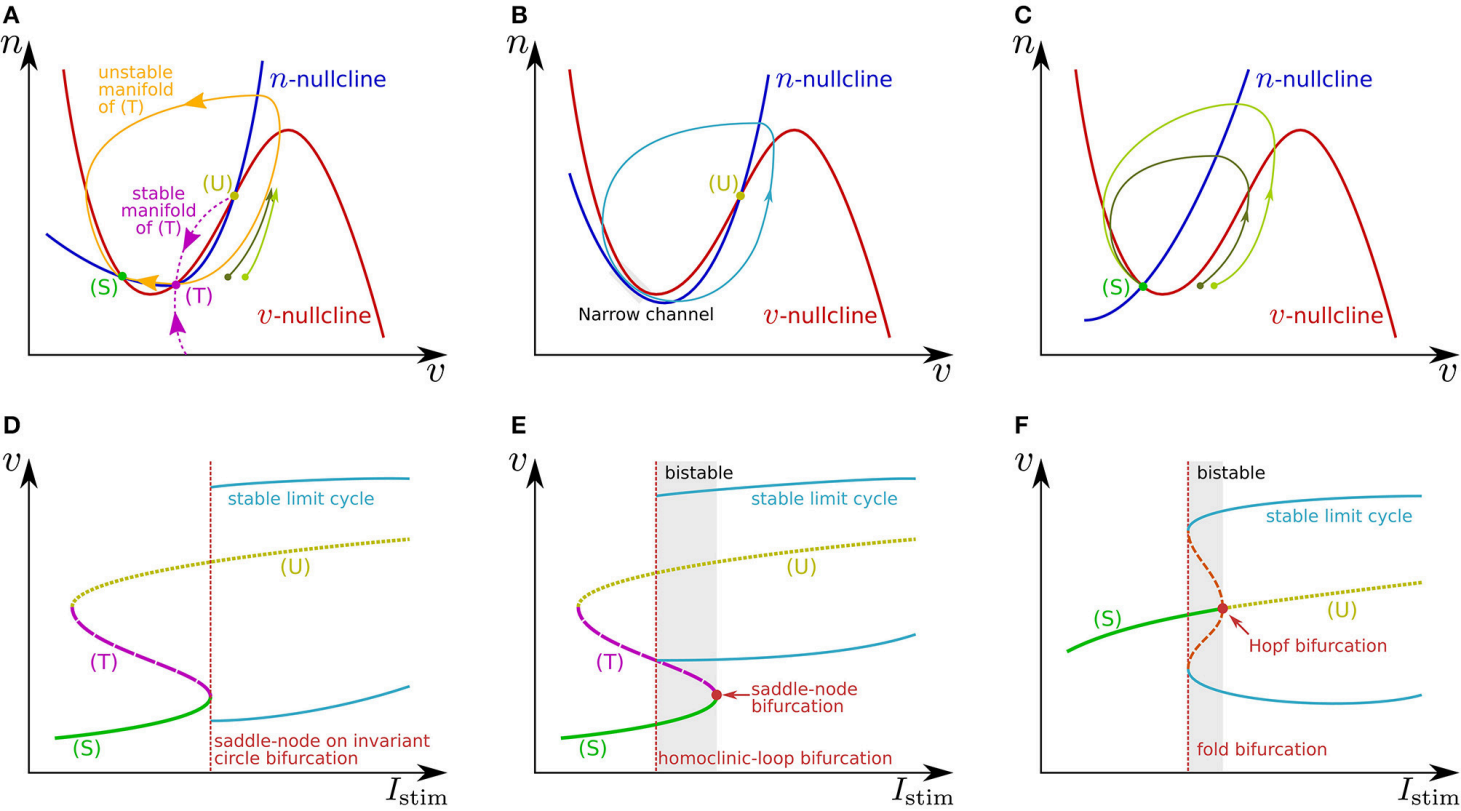
**Illustrations of (A–C) phase planes and (D–F) bifurcation diagrams in the fast subsystem**. The characteristics of neuronal activities can be adjusted by exploiting their mechanisms explained in the main text. For example in **(A)**, the threshold voltage can be increased while the resting membrane potential is held constant if (T) is displaced rightward while (S) is fixed by tuning the slope of the *v*- or *n*-nullcline. The ratio of the time constants of *n* and *v* is one of the factors that determine the shape of the stable and unstable manifolds of (T) and the limit cycle.

It is known that many spike generation systems can be projected onto two-variable systems without critically distorting their dynamics. Typically, the equations of the projected system are in the following form:

(1) $$\begin{array}{rcl} \frac{dv}{dt} & = & f_{v}\left(v,n\right)+I_{\text{stim}}, \end{array}$$

(2) $$\begin{array}{rcl} \frac{dn}{dt} & = & f_{n}\left(v,n\right), \end{array}$$

where *v* is the membrane potential and *n* is a variable that abstractly represents the activity of ionic channels. A stimulus current is represented by *I*_stim_. Figures 1A,C illustrate the phase plane of two typical spike generation systems. The *v*-nullcline, a set of points on which $\frac{dv}{dt}=0$, is N-shaped in both systems, which intersects the *n*-nullcline at three points in (Figure 1A) and at one point in (Figure 1C). In both cases, $\frac{dv}{dt}$ is negative (positive) above (below) the *v*-nullcline, and $\frac{dn}{dt}$ is negative (positive) above (below) the *n*-nullcline.

In Figure 1A, the leftmost intersection (equilibrium) (S) is a stable point, the middle (T) is a saddle point, and the rightmost (U) is an unstable point. Without any perturbation, the state point stays at (S), which corresponds to the resting state. If an excitatory instantaneous pulse stimulus is applied to the system, the system state is displaced horizontally rightward because *v* is the differential voltage of the membrane capacitance to which the stimulus current is directly injected. If the displacement is sufficiently large and the state point crosses the stable manifold of (T), it goes back to (S) along the longer branch of the unstable manifold of (T) by moving upward to the right and then leftward. This temporal increase in *v* is the mechanism of spike generation. Because the trajectory (the orbit of the state point) is attracted to the unstable manifold of (T), the shape of the spikes is not strongly dependent on the stimulus intensity. The threshold voltage for spike generation is determined by the stable manifold of (T) because if the state point does not cross it, the point goes back to (S) along the shorter branch of the unstable manifold by just moving leftward.

The *v*-nullcline is displaced upward by the application of an excitatory sustained stimulus *I*_stim_. Through this transition, (S) and (T) move toward each other, coalesce, and then disappear, which produces a stable limit cycle from the longer branch of the unstable manifold of (T). While the state point stays on the limit cycle, its *v* coordinate repeatedly increases and decreases, which is the mechanism of repetitive spiking. This process, the disappearance of two equilibria and the appearance of a limit cycle, is called a saddle-node on invariant circle bifurcation, and the critical value of *I*_stim_ is a bifurcation point. Figure 1D shows a bifurcation diagram illustrating an overview of this transition. The horizontal axis is for *I*_stim_ (the bifurcation parameter), and the dynamical structure of the phase plane for each value of *I*_stim_ is projected onto the 1-d space of the vertical axis, *v*. Here, the limit cycle is represented by its maximum and minimum values of *v*. As is illustrated in Figure 1B, just above the bifurcation point, the limit cycle passes through a region near both of the *v*- and *n*-nullclines. Because both $\frac{dv}{dt}$ and $\frac{dn}{dt}$ are small in this region, the state point takes a long time to pass, which extends the period time of the limit cycle. As *I*_stim_ is closer to the bifurcation point, this effect is stronger. It extends the period time, which diverges to infinity when *I*_stim_ reaches the bifurcation point. This mechanism accounts for the Class I property in Hodgkin's classification.

If *n* is sufficiently faster than *v*, a stable limit cycle is produced via a homoclinic-loop bifurcation before (S) and (T) coalesce (Figure 1E). As the system is closer to the homoclinic-loop bifurcation point, the period of the limit cycle is extended to infinity by the extended passing time of a region near (T). Because the limit cycle appears before (S) disappears, the system is bistable in the range of *I*_stim_ between the homoclinic-loop and saddle-node bifurcation points.

In Figure 1C, the unique equilibrium (S) is stable, which corresponds to the resting state. If the state point is displaced beyond the rising part of the *v*-nullcline by an excitatory instantaneous pulse stimulus, it starts moving rightward because $\frac{dv}{dt}>0$ below the *v*-nullcline. It then turns to left when it crosses the *v*-nullcline again. This is the mechanism of spike generation in this type of system. The magnitude of the spike, which is the maximum value of *v* on the spike's trajectory, is determined by its intersection with the *v*-nullcline, which is dependent on the starting point to which the state point is displaced by the stimulus. Thus, the spike shape is dependent on the stimulus intensity, which is referred to as graded response.

When *I*_stim_ is a positive sustained stimulus, the *v*-nullcline is displaced upward, by which (S) is transferred upward to the right. At a critical value of *I*_stim_, the stability of this point is reversed via a subcritical Hopf bifurcation. In the bifurcation diagram (Figure 1F), the appearance of a set of stable and unstable limit cycles via another bifurcation, a fold bifurcation, is seen at a smaller value of *I*_stim_. Once (S) loses stability, the state point jumps to the stable limit cycle, and the system starts to spike repetitively. Because there is no dynamical structure that suppresses the velocity of the state point on the stable limit cycle down close to zero, the spiking frequency is always much higher than 0. This accounts for the Class II property in Hodgkin's classification. This system also has bistability composed of the resting state and the stable limit cycle.

### 2.2. System with slow dynamics

**Figure 2:**
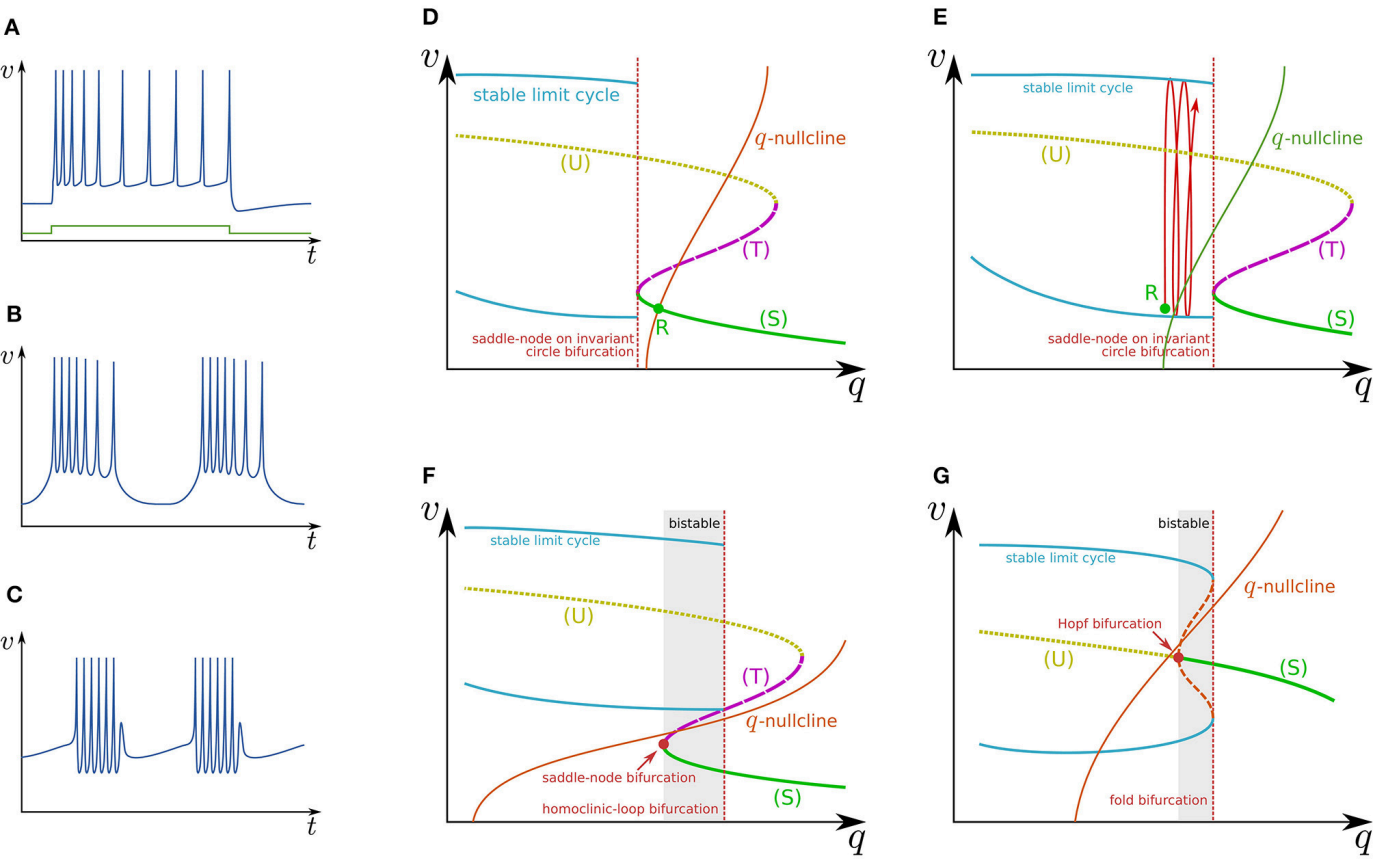
**Illustrations of firing patterns and the dynamical structures that account for their mechanism**. Waveforms of **(A)** RS, **(B)** square-wave bursting, and **(C)** elliptic bursting. The *v*–*q* planes of a simple system that produces the firing patterns of **(D,E)** RS, **(F)** square-wave bursting, and **(G)** elliptic bursting.

Slow hyperpolarizing ionic currents activated by depolarization provide a negative feedback to the spike generation system, which is a most basic mechanism that maintains the spiking behavior “convergent.” These currents play a role as inhibitory stimuli to the spike generation system that modify its dynamical structures. In a case that their time constants are similar, they can be projected even onto a single-variable system. It was elucidated that a simple system composed of a two-variable spike generation subsystem and a single-variable slow subsystem can explain the dynamics of several classes of neuronal activities including RS (Figure 2A), square-wave bursting (Figure 2B), elliptic bursting (Figure 2C), and low-threshold spiking (LTS). In this section, the dynamical structures of the first three classes are explained. Here, a slow subsystem is merged into the spike generation system in the previous section as follows:

(3) $$\begin{array}{rcl} \frac{dv}{dt} & = & f_{v}\left(v,n,q\right)+I_{\text{stim}}, \end{array}$$

(4) $$\begin{array}{rcl} \frac{dn}{dt} & = & f_{n}\left(v,n\right), \end{array}$$

(5) $$\begin{array}{rcl} \frac{dq}{dt} & = & f_{q}\left(v,q\right). \end{array}$$

Figure 2D illustrates the *q*–*v* plane of a system with a Class I spike generation subsystem. Because *q* works as an inhibitory stimulus to the spike generation subsystem, the dynamical structure is dependent on *q* in the opposite manner to *I*_stim_. Thus, the dynamical structure drawn in this figure is similar to a horizontal flip of the bifurcation diagram in Figure 1D. The *q*-nullcline illustrates the characteristics of the slow subsystem; above (below) it, $\frac{dq}{dt}<0$ ($\frac{dq}{dt}>0$). It has an intersection *R* with (S), which is a stable equilibrium point and corresponds to the resting state. In response to an appropriate step stimulus, this system produces RS, which is repetitive spiking whose frequency is initially high and then gradually decreases to a lower value (Figure 2A). Because the dynamical structure of the spike generation subsystem is displaced rightward by an excitatory sustained stimulus (Figure 2E), the state point is released at *R* at the onset of the step stimulus. It is attracted by the stable limit cycle, which is a repetitive spiking state. The slow variable *q* slowly increases because most parts of the limit cycle are above the *q*-nullcline. It converges to a value at which the increase and decrease in the portion of the limit cycle above and below the *q*-nullcline balance. The spiking frequency decreases as *q* increases because the spike generation subsystem is Class I, which produces spikes with a lower frequency in response to a stimulus closer to the bifurcation point. Some types of excitatory cells in the neocortex produce this type of activity. In contrast, some types of inhibitory cells generate faster spikes with a weaker frequency adaptation (fast spiking) (Harris and Shepherd, 2015). Such activity can be modeled by the same model with a weaker adaptation of the spike frequency or a Class II spike generation system.

Figure 2F illustrates the *q*–*v* plane of a system with the spike generation subsystem in Figure 1E. The dynamical structure of the spike generation subsystem is similar to its horizontal flip and the *q*-nullcline has no intersection with any stable states. If the state point is near (S) at some moment, it is attracted to (S). Because (S) is below the *q*-nullcline, *q* slowly decreases, and the state point moves leftward until (S) disappears via the saddle-node bifurcation. The system does not generate any spikes in this phase. The state point is then attracted to the stable limit cycle, which is the only stable state. Because the limit cycle is above the *q*-nullcline, *q* slowly increases, and the state point moves rightward until the limit cycle disappears via the saddle-loop bifurcation. Repetitive firing is produced on the limit cycle in this phase. Then the state point is attracted to (S) again. The system repeats the alternation between the tonic firing and silent phases without any stimuli. This is the mechanism of square-wave bursting (Figure 2B). This class of neuronal activities is involved in life-supporting rhythm generation networks such as a respiratory rhythm generator and a heartbeat rhythm generator (Hill et al., 2001; Negro et al., 2001).

Figure 2G illustrates the *q*–*v* plane of a system with the spike generation subsystem in Figure 1F. As in the previous case, the state point is attracted to (S) if the state point is near to it. Because (S) is below the *q*-nullcline, the state point slowly moves leftward along (S) until it loses stability via the Hopf bifurcation. No spike is generated in this phase. Then the state point is attracted to the stable limit cycle. On the limit cycle, if the increase in *q* above the *q*-nullcline exceeds the decrease below, the state point slowly moves rightward, repetitively generating spikes. After the limit cycle disappears by the fold bifurcation, the state point is attracted to (S) again. The repeated alternation between these two phases is the mechanism of elliptic bursting (Figure 2C). This class of neuronal activities is observed in sleep spindles (Destexhe et al., 1993), which is a characteristic spiking pattern appearing in the thalamus during non-REM sleep.

## 3. Silicon neuron circuits

The core idea of our qualitative-modeling-based approach is to design an ideal silicon neuronal model that reproduces the dynamical structure of a target neuronal class by combination of “device-native” formulae (Kohno and Aihara, 2008b). For low-power analog circuit implementation, the formulae of the *V*–*I* characteristic curves for compact and simple low-power analog circuit blocks can be selected. For digital circuit implementation, polynomials with the lowest order are appropriate because the multiplier is the circuit with the highest cost.

### 3.1. Low-power analog silicon neuron

**Figure 3:**
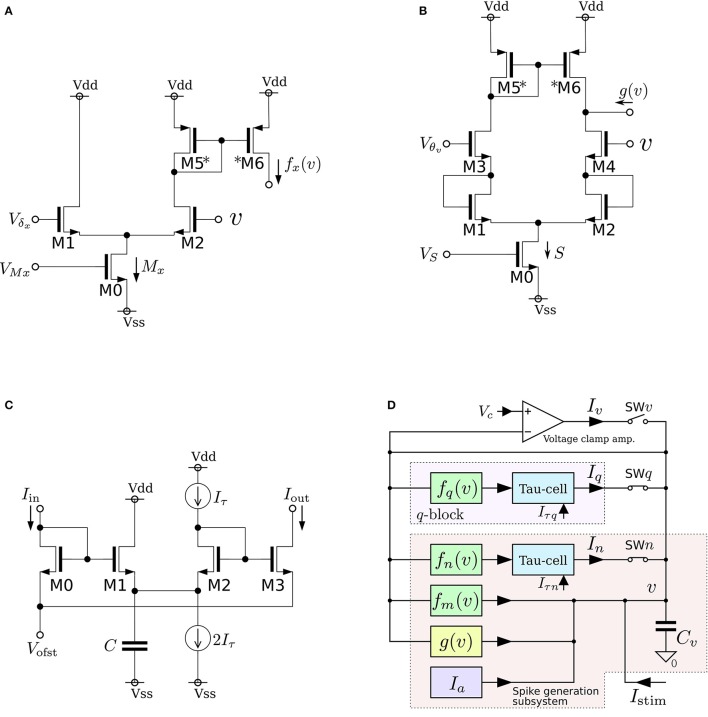
**Circuits of our low-power analog silicon neuron**. **(A–C)** Elemental circuits and **(D)** block diagram. The voltage clamp amplifier placed at the top in the block diagram is used to measure the nullclines experimentally. It is a transconductance amplifier that provides a negative feedback loop to the active terminal of *C*_*v*_. When SW_*v*_ is closed, it locks *v* near *V*_*c*_, which is an input voltage of the amplifier. If SW_*q*_ and SW_*n*_ are open, its output current *I*_*v*_ compensates the current generated by the *f*_*m*_, *g*_*v*_, and *I*_*a*_ circuits. By scanning *V*_*c*_ in an appropriate range and measuring *I*_*v*_, *I*_*n*_, and *I*_*q*_, the *v*-, *n*-, and *q*-nullclines are measured, respectively. The copied outputs of those currents are available for this purpose. This circuit is exploited to find the appropriate parameter voltages that replicate the dynamical structure in the ideal model, which are altered from their ideal values by fabrication mismatch and the transistors' second effects. Reprinted with modification from Kohno et al. (2014b).

We developed a low-power analog SN circuit that can realize the Class I and II neuronal activities in Hodgkin's classification, RS, square-wave bursting, and elliptic bursting. The ideal model of this circuit was designed for implementation by subthreshold MOSFET circuits, which are typically chosen for low-power SN circuits. Because this circuit was intended to be a proof-of-concept for the application of our qualitative-modeling-based approach to integrated circuits, elemental circuits were selected by attaching importance to stability and configurability instead of low-power consumption. The equations of the ideal model are constructed by combining the formulae of the sigmoidal *V*–*I* characteristic curves of differential-pair-based circuits and an integration operation with a leak that can be implemented by the Tau-cell circuit (van Schaik and Jin, 2003). The equations are

(6) $$\begin{array}{rcl} C_{v}\frac{dv}{dt} & = & -g\left(v\right)+f_{m}\left(v\right)-n-q+I_{a}+I_{\text{stim}}, \end{array}$$

(7) $$\begin{array}{rcl} \frac{dn}{dt} & = & \frac{f_{n}\left(v\right)-n}{\tau_{n}}, \end{array}$$

(8) $$\begin{array}{rcl} \frac{dq}{dt} & = & \frac{f_{q}\left(v\right)-q}{\tau_{q}}, \end{array}$$

where *v*, *n*, and *q* represent the membrane potential, the abstracted activity of fast ionic channels, and the abstracted activity of slow ionic channels, respectively. The first two variables compose a fast subsystem, namely the spike generation system, and *q* provides a slow negative feedback to it. Parameters *C*_*v*_, *I*_*a*_, τ_*n*_, and τ_*q*_ are the membrane capacitance, a constant current, and the time constants of *n* and *q*, respectively. Functions *f*_*x*_(*v*) (*x* = *m*, *n*, *q*) and *g*(*v*) are the formulae of the idealized *V*–*I* characteristic curves of the differential pair and transconductance amplifier (Figures 3A,B) as follows:

(9) $$\begin{array}{rcl} f_{x}\left(v\right) & = & M_{x}\frac{1}{1+\exp\left(-\frac{\kappa }{U_{T}}\left(v-\delta_{x}\right)\right)}, \end{array}$$

(10) $$\begin{array}{rcl} g\left(v\right) & = & S\frac{1-\exp\left(-\frac{\kappa }{U_{T}}\left(v-\theta_{v}\right)∕\left(1+1∕\kappa \right)\right)}{1+\exp\left(-\frac{\kappa }{U_{T}}\left(v-\theta_{v}\right)∕\left(1+1∕\kappa \right)\right)}, \end{array}$$

where *U*_*T*_ and κ are the thermal voltage (approximately 26 mV at room temperature) and the capacitive-coupling ratio (approximately 0.7 in our environment), respectively. Parameters *M*_*x*_, δ_*x*_, *S*, and θ are controlled by the externally applied voltages *V*_*M*_*x*__, *V*_δ_*x*__, *V*_*S*_, and *V*_θ_*v*__ in the figures. The *v*-nullcline of this model is given by *n* = *f*_*m*_(*v*) − *g*(*v*) + *I*_*a*_ + *I*_stim_. Because both *f*_*m*_(*v*) and *g*(*v*) are sigmoidal curves and the latter is shallower than the former, it can be *N*-shaped by an appropriate choice of the parameters.

As is drawn in the block diagram of the overall circuit that solves the system equations (Figure 3D), Equation (6) is integrated by *C*_*v*_ whose differential voltage corresponds to *v* and Equations (7) and (8) are solved using the Tau-cell (Figure 3C) whose ideal equation is

(11) $$\begin{array}{rcl} \frac{dI_{\text{out}}}{dt} & = & \frac{I_{\text{in}}-I_{\text{out}}}{CU_{T}∕I_{\tau }}, \end{array}$$

where *C* is the capacitance in the circuit, and *I*_τ_ is a parameter current by which the time constant is arranged. The solutions of Equations (7) and (8) are given by the output current of the Tau-cell circuits (blue boxes in the block diagram) whose input terminals are connected to the outputs of the *f*_*x*_(*v*) circuits (*x* = *n*, *q*). The output current of the lower (upper) Tau-cell, *I*_*n*_ (*I*_*q*_), represents *n* (*q*). Parameter currents *I*_τ*x*_ (*x* = *n*, *q*) and *I*_*a*_ are generated by integrated *V*–*I* converters that are controlled by the externally applied voltages, *V*_τ*n*_, *V*_τ*q*_, and *V*_*a*_, respectively.

**Figure 4:**
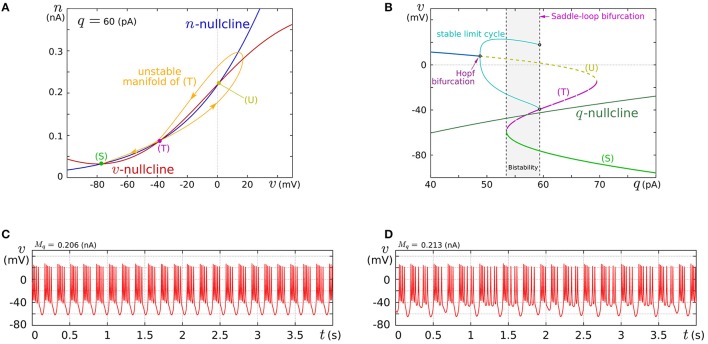
**Example of a dynamical structure and the activities of our low-power analog SN model in the square-wave bursting mode**. **(A)** An example of the *v*–*n* phase plane of the fast subsystem when *q* is fixed at 60 pA. **(B)** Bifurcation diagram of the fast subsystem whose bifurcation parameter is *q*. **(C,D)** Activities of the membrane potential *v*. Reprinted with modification from Kohno and Aihara (2010) (some parameters are modified).

In this review, we focus on the square-wave bursting mode. In this mode, the parameters of the model are selected so that the dynamical structures of the fast subsystem resemble those in Figure 1A. Figure 4A illustrates an example of the *v*–*n* phase plane on which the *v*- and the *n*-nullclines are configured for this mode. The reversed N-shape of the *v*-nullcline is produced by a combination of a rising sigmoidal curve, *f*_*m*_(*v*), and a shallower falling sigmoidal curve, −*g*(*v*). Thus, *M*_*m*_ is increased to make its rising phase steeper and *S* is increased to make its falling phases steeper. These factors control the deepness of its U-shaped and reversed U-shaped regions. The threshold voltage for spike generation depends on the former, which controls the distance between (S) and (T). Because the magnitude of the spikes is suppressed by the latter, the former is generally coordinated to be deeper than the latter to obtain sufficiently high spikes in comparison to the threshold, which can be realized by selecting a smaller value for θ_*v*_ than δ_*m*_. The spike height is also boosted by increasing the time constant of *n*, which slightly increases the spike period as well. The actual spike height can be estimated by drawing the unstable manifolds of (T) whose maximum *v* gives the minimum height. In Figure 4A, the spike height is estimated to be at least 20 mV. In this figure, the longer branch of the unstable manifold of (T) is pulled back to (T) because a relatively small value is selected for the time constant of *n*. In this situation, as described in the previous section, the system undergoes a saddle-loop bifurcation instead of a saddle-node on invariant circle bifurcation in response to the increase in the stimulus current (Figure 1E). Because the slow variable *q* is an inhibitory stimulus current to the *v*–*n* subsystem, this bifurcation structure appears on the *v*–*q* plane in a horizontally flipped manner (Figure 4B).

The appropriate selection of *M*_*q*_ and δ_*q*_ places the *q*-nullcline so that it separates the stable limit cycle and the stable equilibrium (S), which reproduces the dynamical structure in the square-wave bursting illustrated in Figure 2F. The position of the *q*-nullcline relative to the stable states of the fast subsystem is a factor that controls the number of spikes in a burst. This dependence was theoretically elucidated in Wang (1993) using the Hindmarsh–Rose model, which is a qualitative square-wave bursting model. When the slow variable's nullcline is close to the limit cycle, tonic spiking is generated. As it departs from the limit cycle, the tonic spiking becomes chaotic via a period doubling bifurcation cascade. Then regular bursting appears and the number of spikes in a burst decreases one by one down to single-spike bursting. At those transitions, chaotic bursting can be observed if the number of spikes is relatively large. This transition was also observed in an ionic-conductance model of the pancreas β cell (Chay and Rinzel, 1985), in which the maximum conductance of a slow current was swept as the bifurcation parameter. The same transition appears in our circuit model when *M*_*q*_ is swept. This parameter abstractly represents the activity of the slow ionic current which corresponds to the bifurcation parameter in Chay and Rinzel (1985). Examples of regular bursting and chaotic bursting are shown in Figures 4C,D.

**Figure 5:**
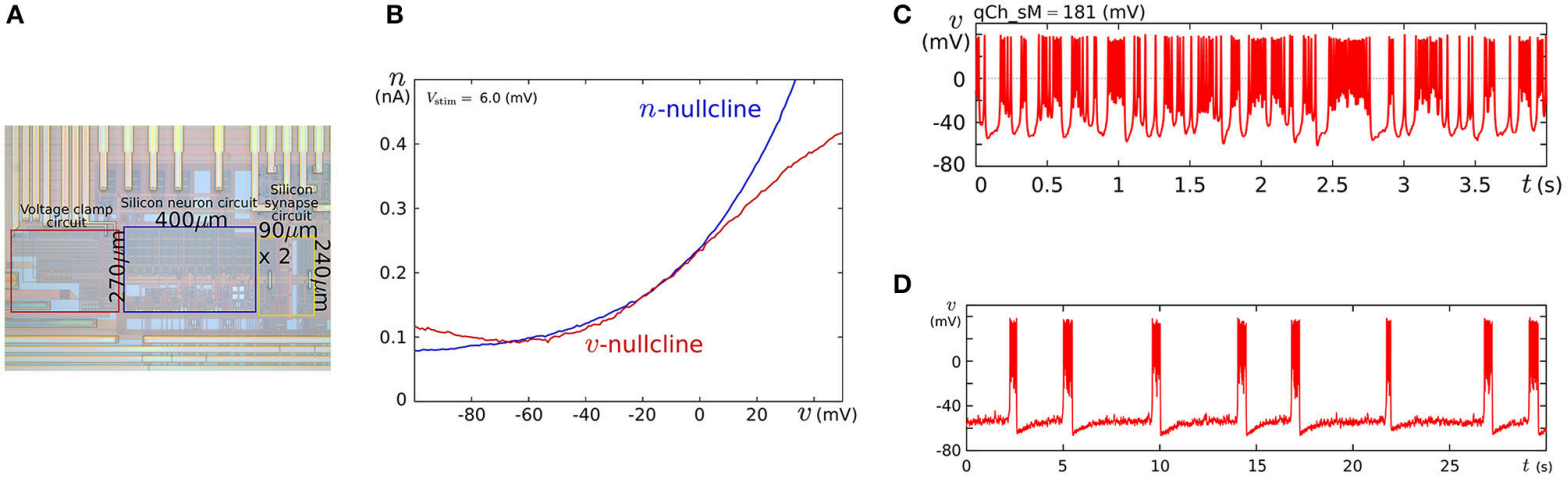
**(A)** Photograph of the fabricated circuit. **(B)** The nullclines measured using the integrated voltage clamp circuit, which resemble those in Figure 4A. Reprinted with modification from Kohno and Aihara (2011). **(C,D)** Square-wave bursting in the circuit. In **(D)**, the time constant of *q* is extended to mimic the activity of the pre-Bötzinger complex bursting neurons. Reprinted with modification from Kohno et al. (2014b). The appropriate parameter voltages for a fabricated circuit are found by iteration in small modification steps. The starting values are calculated by converting the parameters in the ideal model as follows: $V_{M_{x}}=\frac{U_{T}}{\kappa }\text{ ln }\frac{M_{x}}{I_{0N}}$, $V_{S}=\frac{U_{T}}{\kappa }\text{ ln }\frac{S_{v}}{I_{0N}}$, *V*_δ_*x*__ = δ_*x*_, and *V*_θ_*v*__ = θ, where *I*_0*N*_ is the current-scaling parameter of the common-sized NMOS transistors M0 in Figures 3A,B. In accordance with the characteristics of the *V*–*I* converters used to generate *I*_*a*_, *I*_τ*n*_, and *I*_τ*q*_, the voltages *V*_*a*_, *V*_τ*n*_, and *V*_τ*q*_ are calculated, whose detailed equations are omitted here. Then, the modification of the parameter voltages is determined so that the shape and position of the *v*- and *n*-nullclines measured using the integrated voltage clamp amplifier resemble those in the ideal model. In the iteration of this modification, *V*_*M*_*x*__, *V*_δ_*x*__, *V*_*S*_, *V*_θ_*v*__, and *V*_*a*_ (*x* = *m*, *n*) are tuned. After the phase plane structure of the fast subsystem is arranged, *I*_τ*n*_ is modified so that the bifurcation diagram of the fast subsystem in the circuit resembles that in the ideal model. The stable states of the bifurcation diagram can be drawn in circuit experiments by measuring *v* while slowly scanning −*I*_stim_, which is equivalent to *q* from the fast subsystem's viewpoint. In this measurement, SW_*v*_ and SW_*q*_ are opened, and SW_*n*_ is closed. Because *I*_stim_ is generated by an integrated *V*–*I* converter controlled by the voltage input *V*_stim_ and equipped with a copied output of *I*_stim_, this bifurcation diagram can be translated to the *q*–*v* plane. The parameter voltages related to the *q*-nullcline are selected so that the total *q*-*v* plane resembles that of the ideal model. Finally, *V*_τ*q*_ is tuned to obtain bursting activity with the appropriate period.

We fabricated this circuit using a Taiwan Semiconductor Manufacturing Company (TSMC) 0.35 μm mixed-signal CMOS process (Figure 5A). The parameter voltages were tuned following the procedure explained in the caption for Figure 5 on the basis of the dynamical structures in Figure 4. In Figure 5C, a typical bursting activity observed in the circuit experiments is shown. Its activity was always unstable and could not be stabilized by tuning parameters. In Kohno and Aihara (2011, 2013), we pointed out that this fluctuated behavior arises from the intrinsic dynamical structure of square-wave bursting, i.e., the sensitivity to the initial conditions near the saddle-loop bifurcation. By extending the time constant of *q* (decreasing *I*_τ*q*_), we could obtain a bursting pattern with a longer period that is similar to the activity of autonomous bursting cells in the pre-Bötzinger complex that generate the respiratory rhythm (Negro et al., 2001). In this case, the bursting activity is more stable than that in (c) because the extended time constant of *q* makes the trajectory of the state point pass closer to the stable equilibrium (S) and its sojourn time near the saddle-loop bifurcation point shorter. In addition to square-wave bursting, we could also realize Class I and II, RS, and elliptic bursting. For any settings, the power consumption of this circuit including the bias-voltage generators for the Tau-cell circuits did not exceed 72 nW.

### 3.2. Ultralow-power analog silicon nerve membrane

**Figure 6:**
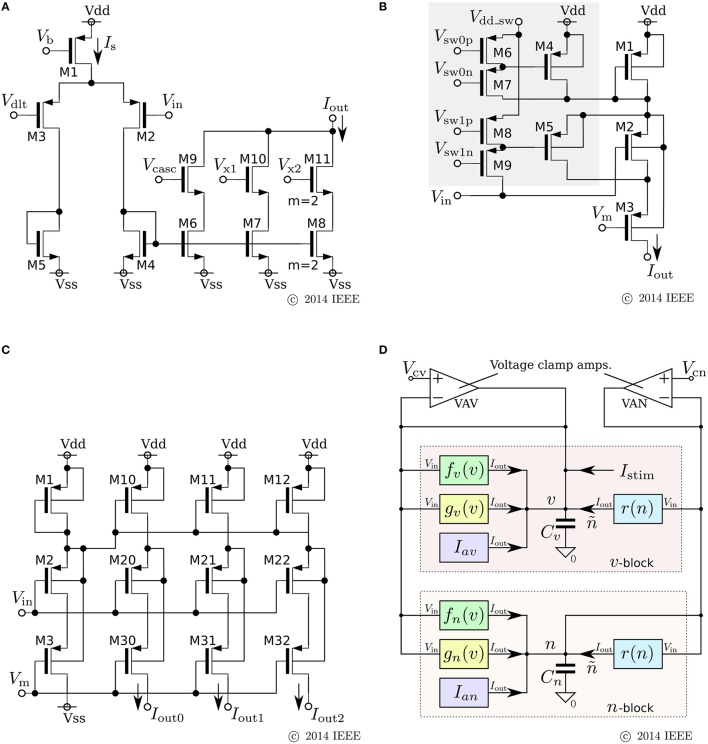
**Circuits of our ultralow-power analog silicon neuron**. **(A–C)** Elemental circuits and **(D)** block diagram. In Equations (30)–(34), *I*_0*P*_ is the current-scaling parameter of common-sized PMOS transistors [M1 in **(A)**, M1 to M3 in **(B)**, and all of the transistors in **(C)**]. Parameter *M*_*x*_ is the drain current of M1 in **(A)**, which is controlled by *V*_b_. Parameters δ_*x*_ (*x* = *v*, *n*) and θ_*x*_ (*x* = *v*, *n*, *r*) correspond to Vdd - *V*_dlt_ and Vdd - *V*_m_ (Vdd = 1.0 V), respectively. Parameter *R*_*x*20_ is 1 (0.5) when M6 is on (off) and M7 is off (on) in panel **(B)**. Parameter *R*_*x*21_ is 1 (2) when M8 is on (off) and M9 is off (on). These two parameters are used to shift the curve of *g*_*x*_(*v*) horizontally. The two voltage clamp amplifiers, VAV and VAN, in **(D)** are for drawing the *v*- and *ñ*-nullclines and evaluating *r*′(*n*) in Equations (36) and (38). The output of these transconductance amplifiers is 0 when the two input voltages are the same and positive (negative) when the “+” input voltage is higher (lower) than that of the “−” input. When they are activated and the *r*_*n*_(*n*) block is deactivated, their outputs are equal to the sums of *f*_*x*_(*v*), *g*_*x*_(*v*), and *I*_*ax*_ (*x* = *v*, *n*). By scanning *V*_cv_ and maintaining *V*_*cn*_ constant, the nullclines are measured. Similarly, when the *r*_*n*_(*n*) block is activated and the other blocks are deactivated, the output of VAN is equivalent to *r*_*n*_(*n*). By scanning *V*_cn_, the dependence of *r*_*n*_(*n*) on *n* is measured, from which *r*′(*n*) can be evaluated. Reprinted with modification from Kohno and Aihara (2014a).

The power consumption of the above circuit is one order of magnitude higher than low-power-oriented leading-edge circuits (Basu and Hasler, 2010; Brink et al., 2013b; Qiao et al., 2015). We developed SN circuitry to attain a lower power consumption that is comparable to these works. A two-variable model that supports the Class I and II neuronal activities was designed on the basis of this circuitry to evaluate its practicality (Kohno and Aihara, 2014a). Its ideal model is given by

(12) $$C_{v}\frac{dv}{dt}=f_{v}(v)-g_{v}(v)+I_{av}-r(n)+I_{\text{stim}},$$

(13) $$\begin{array}{rcl} C_{n}\frac{dn}{dt} & = & f_{n}\left(v\right)-g_{n}\left(v\right)+I_{an}-r\left(n\right), \end{array}$$

where *v* and *n* represent the membrane potential and the abstracted activity of fast ionic channels, respectively. Parameters *C*_*v*_ and *C*_*n*_ are the capacitances that are determined at circuit fabrication, *I*_*av*_ and *I*_*an*_ are parameter currents, and *I*_stim_ is the stimulus current. Functions *f*_*x*_(*v*), *g*_*x*_(*v*), and *r*(*n*) (*x* = *v*, *n*) are monotonic increasing sigmoidal functions that correspond to the idealized *V*–*I* characteristic curves of the elemental circuits shown in Figures 6A–C. Their equations are

(14) $$\begin{array}{rcl} f_{x}\left(v\right) & = & \frac{M_{x}}{1+\exp\left(-\frac{\kappa }{U_{T}}\left(v-\delta_{x}\right)\right)}, \end{array}$$

(15) $$\begin{array}{rcl} g_{x}\left(v\right) & = & I_{0\text{P}}\sqrt{\frac{R_{x20}\exp\left(\frac{\kappa }{U_{T}}\theta_{x}\right)}{1+R_{x21}\exp\left(-\frac{\kappa }{U_{T}}\left(v-\theta_{x}\right)\right)}}, \end{array}$$

(16) $$\begin{array}{rcl} r\left(n\right) & = & I_{0\text{P}}\sqrt{\frac{\exp\left(\frac{\kappa }{U_{T}}\theta_{r}\right)}{1+\exp\left(-\frac{\kappa }{U_{T}}\left(v-\theta_{r}\right)\right)}}. \end{array}$$

The parameters in these equations are explained in the caption for Figure 6. A block diagram of a circuit that solves this model is shown in Figure 6D. In this circuit, *v* and *n* are coded by the voltage difference between Vdd and the voltage across capacitors *C*_*v*_ and *C*_*n*_, respectively. Blocks *f*_*x*_(*v*) and *g*_*x*_(*v*) (*x* = *v*, *n*) correspond to the circuits in (a) and (b), respectively. For *g*_*v*_(*v*), the transistors in the shadowed region are omitted to simplify the circuit. The two blocks of *r*(*n*) correspond to a single circuit of (c).

Equations (26) and (28) are transformed as follows by defining the variable *ñ* ≡ *r*(*n*):

(17) $$\begin{array}{rcl} C_{v}\frac{dv}{dt} & = & f_{v}\left(v\right)-g_{v}\left(v\right)+I_{av}-ñ+I_{\text{stim}}, \end{array}$$

(18) $$\begin{array}{rcl} C_{n}\frac{dñ}{dt} & = & r^{\prime }\left(n\right)\left(f_{n}\left(v\right)-g_{n}\left(v\right)+I_{an}-ñ\right). \end{array}$$

By using the two voltage clamp amplifiers, VAV and VAN, in the block diagram, the *v*- and *ñ*-nullclines are measured and *r*′(*n*) is evaluated (see the caption for Figure 6).

The major improvement of this circuitry from the previous SN is a reduction in the static current consumption. In the *f*_*x*_(*v*) circuit, M7, M8, M10, and M11 are used to extend the output current range without increasing the tail current *I*_*s*_. The current consumption of the cascode circuitry in *g*_*x*_(*v*) is equal to the output current, whereas the transconductance amplifier in the previous *g*_*x*_(*v*) circuit constantly consumes its maximum current. The integration of *n* is performed by a capacitor instead of the Tau-cell. The Tau-cell is an easy-to-use current-mode integrator with a constant time constant, which supports a wide range of input and output currents. However, it requires additional circuits that generate *I*_τ_, 2*I*_τ_, and *V*_ofst_. The currents required to drive these circuits are cut off by the direct integration of the currents into a capacitor. In this case, the acceptable range of the variables is limited by the range of *r*_*n*_(*n*), on which the time constant of *n* is dependent. The above nullcline-drawing function helps to find the appropriate parameter values under these limitations; once *r*_*n*_(*n*) is specified by the time constant requirement of *n*, the appropriate dynamical structure for the target neuronal class can be constructed within the acceptable range of the variables by tuning the other parameters utilizing the nullcline drawing function.

**Figure 7:**
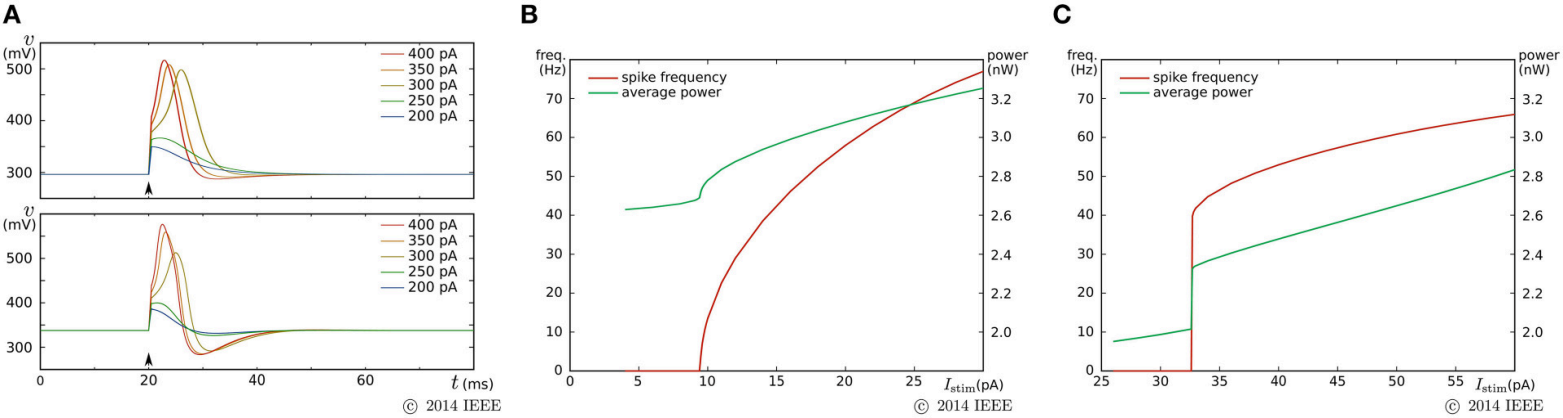
**Circuit simulation results of our ultralow-power analog silicon neuron**. **(A)** Responses to a pulse stimulus in the Class I mode (upper) and the Class II mode (lower). **(B,C)** Spiking frequency and power consumption in response to a sustained stimulus. The horizontal axes represent the intensity of the stimulus. **(B)** is for the Class I mode and **(C)** is for the Class II mode. Reprinted with modification from Kohno and Aihara (2014a).

By a similar parameter tuning procedure to that for the fast subsystem in the previous SN circuit, we found the parameter values for the Class I and II modes. Figure 7 shows the simulation results obtained using the Spectre circuit simulator with the TSMC 0.25 μm mixed-signal CMOS process development kit. Capacitances *C*_*v*_ and *C*_*n*_ are implemented by metal–insulator–metal capacitors (MIMCAPs) with capacitances of 1.5 and 2.0 pF, respectively. In the pulse stimulus responses (Figure 7A), the height of spike is dependent on the intensity of the stimulus (graded response) in the Class II mode (lower plot), whereas the dependence is weak in the Class I mode (upper plot). In the sustained stimulus response (Figures 7B,C), the spike frequency can be reduced close to 0 by applying a sufficiently weak stimulus in the Class I mode [(B)], whereas spike generation is terminated before the spike frequency reaches close to 0 in the Class II mode [(C)]. In both settings, the power consumption increases with the spike frequency and is less than 3.2 nW when the spike frequency is less than 70 Hz.

### 3.3. A silicon neuronal network by digital arithmetic circuits

Generally, the power consumption of a dynamical digital circuit is higher than that of the subthreshold analog circuits used in the previous sections. However, the continuous evolution of the fabrication process is lowering the power consumption. In Merolla et al. (2014), a combination of ultrafine processes and technologies such as asynchronous and near-threshold logic realized low-power silicon neuronal networks whose power consumption per neuron is only one order of magnitude higher than the lowest-power analog silicon neurons. Reduced power consumption these days is facilitating a fascination with the scalability and stability of digital circuits.

**Figure 8:**
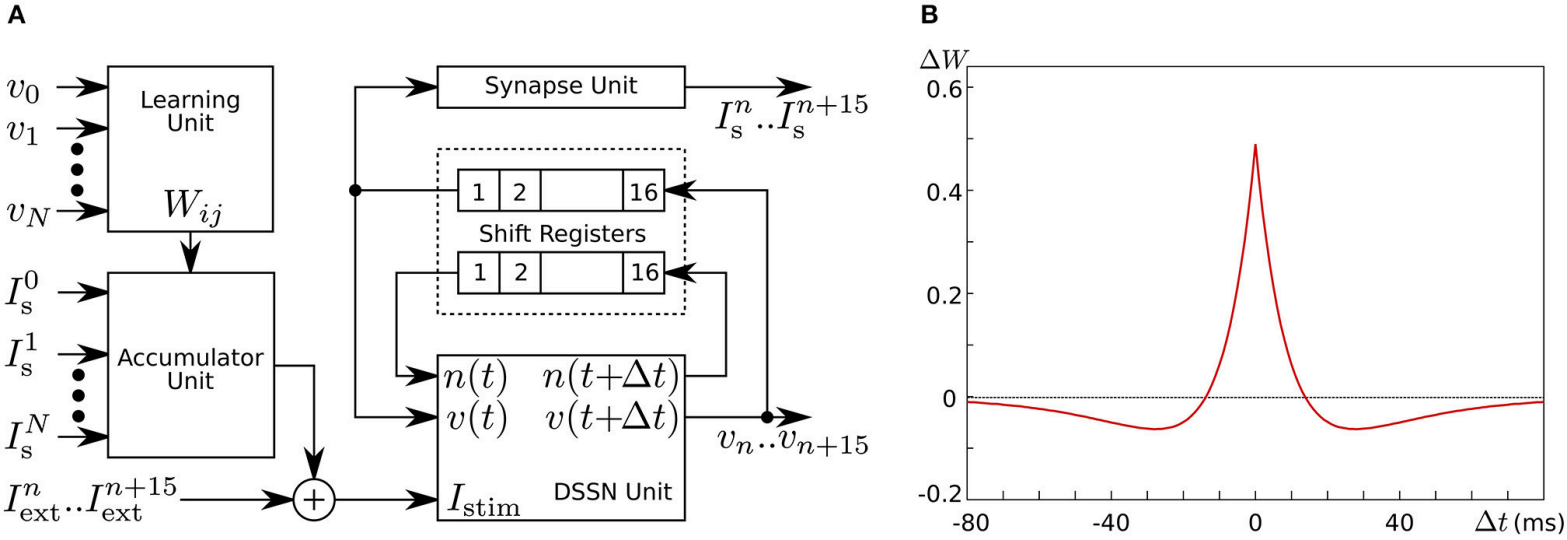
**(A)** Block diagram of our silicon neuronal network module, which solves the equations for 16 SNs. **(B)** Mexican-hat-type spike-timing-dependent learning curve utilized in the Hebbian-type learning in our digital silicon neuronal network. Reprinted with modification from Li et al. (2013).

In Li et al. (2012, 2013), we developed a silicon neuronal network in an FPGA based on our qualitative-modeling-based silicon neuronal model for digital arithmetic circuits (Kohno and Aihara, 2007). Figure 8A is a block diagram of its basic unit, the silicon neuronal network module (SNNM), which executes a calculation related to 16 SNs including spike-timing-dependent learning. Larger-scale networks are constructed by connecting more than one of these modules in parallel.

The digital spiking silicon neuron (DSSN) Unit calculates the silicon neuronal model designed with the same principle as above so that it can be implemented with reduced hardware resources by using the minimum number of multipliers. Its equations are given by

(19) $$\begin{array}{rcl} \frac{dv}{dt} & = & \frac{\phi }{\tau }\left(f\left(v\right)-n+I_{0}+I_{\text{stim}}\right), \end{array}$$

(20) $$\begin{array}{rcl} \frac{dn}{dt} & = & \frac{g\left(v\right)-n}{\tau }, \end{array}$$

(21) $$f(v)=\{\begin{array}{ll} a_{fn}{\left(v-b_{fn}\right)}^{2}+c_{fn} & (v<0),\\ a_{fp}{\left(v-b_{fp}\right)}^{2}+c_{fp} & (v\geq 0), \end{array}$$

(22) $$g(v)=\{\begin{array}{ll} a_{gn}{\left(v-b_{gn}\right)}^{2}+c_{gn} & (v<r_{g}),\\ a_{gp}{\left(v-b_{gp}\right)}^{2}+c_{gp} & (v\geq r_{g}), \end{array}$$

where *v* and *n* are variables with no unit that represent the abstracted membrane potential and ionic current activity, respectively. Parameters ϕ and τ configure the time scale of the model's activity. A stimulus input is represented by *I*_stim_. The other parameters determine the shape of the nullclines on the *v*–*n* phase plane; *n* = *f*(*v*)+*I*_0_ for the *v*-nullcline and *n* = *g*(*v*) for the *n*-nullcline. The *N*-shaped *v*-nullcline is realized by the piecewise quadratic function *f*(*v*) instead of a cubic function, which reduces the number of multiplications between variables. Multiplication between a constant and a variable can be implemented by small numbers of shifters and adders if the number of active bits in the constant's binary expression is small. By the parameter tuning procedure similar to our analog SNs, parameter sets that realize the Class I and II activities were found with which this model is solved by Euler's method with fixed-point operations.

The Synapse Unit calculates the following silicon synapse model by Euler's method with fixed-point operations.

(23) $$\frac{dI_{\text{s}}}{dt}=\{\begin{array}{ll} \alpha (1-I_{\text{s}}) & \text{when }v\geq 0,\\ -\beta I_{\text{s}} & \text{when }v<0, \end{array}$$

where *I*_*s*_ is the post-synaptic stimulus received by other SNs. Parameters α and β determine the time constants of *I*_s_ in the rising and falling phases, respectively. This model was developed on the basis of the kinetic models of chemical synapses (Destexhe et al., 1998) so that it can transmit the analog information of the graded responses in Class II neurons.

The Accumulator Unit calculates the sum of *I*_*s*_ given by other SNNMs as follows:

(24) $$\begin{array}{rcl} I_{\text{ss}}^{j} & = & c\underset{i}{\sum }W_{ji}I_{\text{s}}^{i}, \end{array}$$

where *c* is a scaling parameter, *W*_*ji*_ is the synaptic weight from SN *i* to SN *j*, and $I_{\text{s}}^{i}$ is *I*_s_ generated by the Synapse Unit for neuron *i*. The sum of $I_{\text{ext}}^{j}$ and $I_{\text{ss}}^{j}$ is given to the SN's stimulus input, *I*_stim_.

The Learning Unit supports spike-time-dependent learning with exponential-based rules. In Li et al. (2013), we implemented the following Hebbian-type rule:

(25) $$\begin{array}{rcl} \Delta W_{ji} & = & A_{+}\exp\left(\frac{-|\Delta t_{ji}|}{\tau_{+}}\right)-A_{-}\exp\left(\frac{-|\Delta t_{ji}|}{\tau_{-}}\right), \end{array}$$

where Δ*W*_*ji*_ is the modification applied to *W*_*ji*_, and Δ*t*_*ji*_ is the time between the two nearest spikes of neuron *j* and neuron *i*. The time of a spike here is defined as the time when *v* exceeds 0. Parameters *A*_+_ and *A*_−_ configure the amplitude of the learning curve and τ_+_ and τ_−_ specify its time scale. Mexican-hat-type learning curves can be realized by selecting appropriate parameter values (Figure 8B).

**Figure 9:**
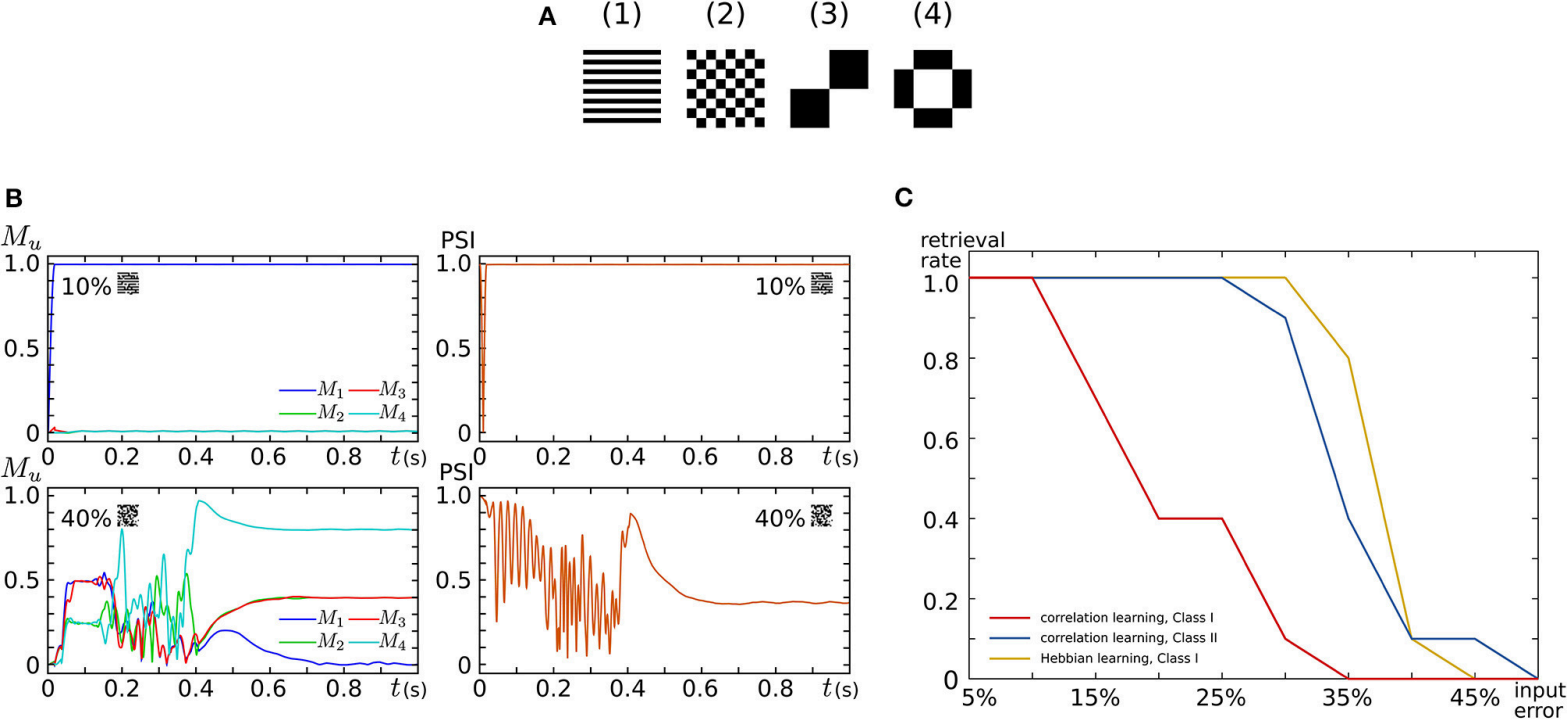
**Auto-associative memory tasks executed in our all-to-all digital silicon neuronal networks**. **(A)** Stored patterns. Each pattern is composed of 256 (16 × 16) pixels with a value of 1 or −1. Black (white) pixels have a value of 1 (−1). Reprinted with modification from Li et al. (2012). **(B)** Examples of the transition of *M*_*u*_ and PSI when the input pattern has 10 and 40% flipped pixels. Reprinted with modification from Li et al. (2012). **(C)** Error recovery performance when the patterns are stored by correlation learning and the SNs are in the Class I mode (red) and in the Class II mode (blue). The yellow plot is for the case where the patterns are stored by Hebbian-type spike-timing-dependent learning and the SNs are in the Class I mode. The horizontal axis is the ratio of the flipped pixels to the total number of pixels (the error level). The vertical axis represents the rate of successful retrieval rate. For each error level, 10 trials were executed. Reprinted with modification from Li et al. (2013).

By implementing 16 SNNMs in an FPGA chip, we constructed an all-to-all connected silicon neuronal network composed of 256 SNs. To verify its functionality, we executed associative memory tasks in which the four patterns shown in Figure 9A are stored. A pattern comprises 256 (16 × 16) pixels, each of which has a value of 1 or −1. In the figure, a black (white) pixel has value of 1 (−1). Firstly, these patterns were stored by correlation learning without using the Learning Unit as follows:

(26) $$W_{ij}=\{\begin{array}{ll} \frac{1}{4}\sum_{u=1}^{4}x_{i}^{u}x_{j}^{u} & \text{when }i\neq j,\\ 0 & \text{when }i=j, \end{array}$$

where $x_{i}^{u}$ represents the value of the *i*th pixel in pattern *u*. In the retrieval process, all SNs are repetitively spiking owing to the application of an appropriate sustained stimulus $I_{\text{ext}}^{i}$ for all *i*. Their initial spiking phases are arranged by a short positive external input applied before the sustained stimulus only to the SNs that correspond to the pixel with a value of 1 in the input pattern. The input patterns were generated by flipping the values of randomly selected pixels in a stored pattern. Figure 9B plots examples of observed retrieval processes, in which the time step for numerical integration is 375 μs. In the left column, *M*_*u*_, an index that reflects the correlation between the current spiking pattern and the *u*th pattern, is plotted. This value is 0 when the spiking pattern has no relation with the *u*th pattern and approaches 1 as the pattern matches to it. In the right column, the phase synchronization index (PSI) that reflects the degree of synchronization is plotted. It is 0 when the SNs are spiking fully asynchronously and approaches 1 as their spike timing is synchronized. In the upper row, 10% of the pixels in pattern (1) are flipped and applied as inputs. In this case, *M*_1_ quickly increases and remains near 1, which indicates the successful retrieval of pattern (1). In the lower row, 40% of the pixels are flipped. Then, none of the values of *M*_*u*_ remain close to 1, which indicates that no pattern was retrieved. The PSI plotted in the right column stayed near 1 when a correct pattern was retrieved but not when no pattern was retrieved. We executed 100 retrieval processes: 10 different levels of flipped (error) pixels from 5 to 50% in 5% increments and 10 patterns for each error level. The red and blue plots in Figure 9C show the rate of successful retrieval when the SNs are in the Class I and II modes, respectively. The network could retrieve a correct pattern from a larger number of errors when the SNs are in the Class II mode than when they are in the Class I mode. This indicates that the spiking dynamics may play important roles in auto-associative memory tasks.

Second, the four patterns were stored by activating the Learning Unit as follows. The stored patterns were applied to the network in the sequence of (1), $\bar{\left(1\right)}$, (1), $\bar{\left(1\right)}$, .. (2), $\bar{\left(2\right)}$, .. (3), $\bar{\left(3\right)}$, .. (4), $\bar{\left(4\right)}$, .. (1), $\bar{\left(1\right)}$, .., where $\bar{\left(n\right)}$ means the reversed pattern of (*n*). A couple of a stored pattern and its reversed pattern was repeated eight times in a block. This sequence was applied until one of the values of *W*_*ij*_ reached 1 or -1 by the modification in Equation (26). In the retrieval process, the Learning Unit was deactivated and input patterns with errors were applied in the same way as above. The yellow plot in Figure 9C shows the rate of successful retrieval when the SNs are in the Class I mode. The error recovery performance exceeded both results with correlation learning. In our preliminary results with SNs in the Class II mode, this performance was further boosted (not shown in the figure). The spiking dynamics may also be important in auto-associative memory with spike-timing-dependent learning rules.

## 4. Discussion

As reviewed above, our silicon neuron circuits can realize different classes of neuronal activities by selecting appropriate parameter values and their characteristics can be modified by finely tuning the parameters as shown in Figure 5D. This high configurability is advantageous not only for bio-silico hybrid systems but also for constructing “ field-programmable” silicon neuronal networks in which each SN can be reconfigured after fabrication or each SN autonomously obtains appropriate dynamical properties on the basis of the history of stimulus inputs as in the brain. This high configurability arises from the fact that the activity of many neuronal classes can be explained using common dynamical structures that are reproduced in our models by a combination of implementation-efficient formulae. In contrast, the circuitry is simplified by supporting only one neuronal class in the non-I&F-based SN circuits developed by a similar approach (Basu and Hasler, 2010; Brink et al., 2013b). These circuits realize ultralow power consumption down to several nanowatts at the expense of configurability. In their SN network systems, the configurability is supplemented by accommodating a sufficiently large SN circuit pool, in which the appropriate SNs for a desired network are activated. Our circuit in Section 3.2 supports both Class I and II neuronal activities and consumes a similar power; however, it has the drawback of high configurability. The circuit has to be configured appropriately by tuning a number of parameter values, and additional circuits are required for storing parameter values. The complexity of the configuration process is solved by parameter tuning procedures that utilize the nullcline drawing circuits as explained in detail in Section 3.1. This procedure is still not straightforward, but all of the students who worked on our circuit learned to be able to finish the tuning procedure within several tens of minutes. For a large-scale silicon neuronal network, this procedure has to be automated. It may be done by metaheuristic approaches similar to those utilized in Grassia et al. (2011). The power consumption and area occupied by additional circuits for storing parameter values may be reduced by evolving non-volatile memory technologies such as memristors.

In digital silicon neuronal networks, the accumulation of synaptic inputs consumes a considerably larger amount of hardware resources than SN circuits. Thus, the compactness of the SN circuit is not a major issue. The advantage of our circuit is that its model is non-I&F-based and thus can mimic the spike-generation-related properties in neuronal activities more finely than I&F-based circuits. One of these properties is the graded response in Class II neurons. Because the graded response is found in the brain, as mentioned in the introduction, there is possibility that it plays some roles in information processing in the brain. Our silicon neuronal network model intends to provide a platform in which a wide variety of neuronal activities including the dynamics of spike generation is qualitatively reproduced without a major increase in hardware resource consumption. For this goal, our SN model is being expanded so that it can realize more classes of neurons including RS, LTS, and IB as well as autonomous bursting supported by our analog SN. It has four variables (two original and two additional slow variables) but still can be solved by one multiplication per a numerical integration step. The details of this model is explained in Nanami and Kohno (2016).

A goal of our analog silicon neuronal circuits is to establish an ultralow-power general-purpose silicon neuronal network platform that will be applicable to neuromimetic computing when the mechanism of information processing in the nervous system is elucidated. We expect that it has an advantage also in the application to large-scale neuronal network simulators (Schemmel et al., 2010; Stromatias et al., 2013) and brain-prosthetic devices such as an artificial hippocampus (Berger et al., 2012; Hampson et al., 2013; Song et al., 2015), an artificial cerebellum (Hogri et al., 2015), and an artificial prefrontal cortex (Hampson et al., 2012) because our circuits meet their requirements of a low power consumption and the ability to mimic various complex neuronal activities finely. Construction of such systems may contribute to the elucidation of the brain's mechanisms by the “analysis by synthesis” approach. Our digital silicon neuronal network platform is also applicable to neuromimetic computing and large-scale neural network simulation. It consumes more power than analog circuits but has advantage in scalability.

## Author contributions

MS performed circuit experiments on analog silicon neurons. JL implemented, simulated, and performed experiments on digital silicon neuronal networks. TN expanded the digital silicon neuron model. KA checked the mathematical correctness of the research. TK did all other things.

## Funding

The works reviewed in this article were partially supported by the JST PRESTO program, the JST CREST program, and a JSPS Grant-in-Aid for scientific Exploratory Research Number 25240045.

### Conflict of interest statement

The authors declare that the research was conducted in the absence of any commercial or financial relationships that could be construed as a potential conflict of interest.
